# An overview of basic pathophysiological interactions between gut bacteria and their host

**DOI:** 10.3389/fnut.2025.1565609

**Published:** 2025-08-12

**Authors:** Mathilde Simonson, Thomas Simonson, Estelle Nobécourt

**Affiliations:** ^1^Department of Endocrinology, Diabetes and Nutrition, GHSR, Centre Hospitalo-Universitaire de la Réunion, Saint-Pierre, La Réunion, France; ^2^Université de La Réunion, INSERM, UMR 1188 Diabète Athérothrombose Thérapies Réunion Océan Indien (DéTROI), Plateforme CYROI, Saint-Denis, France; ^3^Laboratoire de Biologie Structurale de la Cellule (CNRS UMR 7654), Ecole Polytechnique, Institut Polytechnique de Paris, Palaiseau, France

**Keywords:** gut microbiome, metabolite, gene regulation, nutrition, inflammation, small chain fatty acid, bile acid

## Abstract

Sometimes referred to as a “forgotten organ,” the gut microbiome (GMB) of humans includes hundreds of commensal bacterial species, which carry several million genes and complement our physiology. Commensal bacteria break down indigestible dietary fiber and provide essential metabolites. These often have a dual action: as “stand-alone” chemicals (e.g., combustibles or emulsifiers) and as signaling molecules that influence host gene expression and physiology. A second function of gut bacteria is to help maintain the intestinal barrier, partly by conditioning the host immune system. Alteration and damage to the GMB have been linked to many pathologies. This review provides an introduction to the more basic mechanisms of GMB-host interaction. It focuses on (a) gut bacteria and their metabolites, and (b) the metabolites’ role in host gene regulation and homeostasis. To this end, recent articles were selected, along with some earlier ground-breaking articles. We consider microbiome composition and intestinal homeostasis, microbiome composition and dysbiosis, immune modulation, gut bacteria metabolite chemistry and host gene regulation.

## 1 Introduction

Our body contains more microbes than human cells: around 10^14^ microbes vs. 3 10^13^ host cells. Microbes represent therefore on the order of 1% of our mass: roughly a kilogram. They include bacteria, archaea, and fungi, and form what is known as the human microbiome. The most abundant are bacteria that inhabit the intestine, or gut: around 4 10^13^ bacterial cells. Sometimes referred to as a “forgotten organ,” the gut microbiome (GMB) includes hundreds of bacterial species and thousands of strains, which carry several million genes (1, 2): far more than the human genome. While microbe diversity in the unborn fetus is low, the GMB develops immediately after birth. Human adults harbor a GMB with a signature composition, largely stable over time (3, 4) outside of specific perturbations (like disease) or development stages (like pregnancy). Within an ethnic group, GMB differences between individuals can be due to old age, nutrition, disease or antibiotic treatments (5–8). Alteration and damage to the GMB have been linked to many pathologies, including obesity, diabetes, inflammatory bowel diseases (IBD) and digestive cancers, as well as cardiovascular and mental diseases (9).

Most bacteria in the human gut are not pathogenic or parasitic but “commensal.” Their population density is largest by far in the large intestine, or colon. They form a mutually beneficial relationship with their host, and are essential for many functions. A first set of functions involve extracting energy and chemicals from our diet, making them active participants in our nutrition. Thus, germ-free mice that have never encountered microbiota put on less fatty mass (10). Commensal bacteria help break down indigestible dietary fiber, then provide essential metabolites, such as short chain fatty acids (SCFAs), secondary bile acids (BAs), and assorted vitamins and hormones (11). Indeed, over 10% of metabolites in host systemic circulation are estimated to originate from gut microbiota (12). These metabolites often have a dual action: as “stand-alone” chemicals and as signaling molecules (13) that influence host gene expression. Thus, bile acids emulsify and transport fatty nutrients, and can also bind and activate several transcription factors (14). Several SCFAs are burned as fuel by colonic epithelial cells, and can also perform cell signaling by activating specific G-Protein Coupled Receptors (15). The interplay between the GMB and host gene activity is illustrated by the digestive tract of germ-free (GF) mice, where one observes increased expression of genes involved in ileal (small intestine) lipid metabolism, oxidative stress control and gluconeogenesis (16). The lack of a GMB is associated with sweeping metabolic changes, including increased energy requirements and anaerobic glycolysis (17–20).

A second function of gut bacteria is to help maintain the intestinal barrier, partly by participating in the development of the host immune system in both the large and small intestine. Indeed, the gut barrier is a key interface in our body, and home to most of our immune cells. It must intercept and combat pathogenic bacteria while tolerating commensal ones. Commensals help strengthen and maintain the barrier, contributing for example to mucus production, energy homeostasis of intestinal epithelial cells, and the integrity of the inter-cellular junctions between them (21). Commensals occupy space that could otherwise be colonized by pathogens, and secrete anti-bacterial substances (22). They also participate in the immune development of newborns. Thus, in GF mice, transcriptomic changes at the gut barrier relative to ordinary mice are mostly associated with host immune functions (16).

The GMB thus has a major impact on health and physiology, and has generated enormous interest over the last decade, with thousands of articles published, including many reviews. The goal of the present review is to provide an introduction to the more basic mechanisms of GMB-host interaction. It does not consider gut archaea, eukaryotes, or viruses. Rather, it focuses on the major GMB component: gut bacteria, their metabolites, and their role in gene regulation and homeostasis of the host. The review is based on a selection of articles from the very recent literature, along with some earlier ground-breaking articles. There are four sections below. The first considers GMB composition and intestinal homeostasis. It recalls the basic physical structures and cell types in the gut, and summarizes the main information on gut bacterial genomics and taxonomy, including the diversity and variability within individual hosts (alpha-diversity) and within a host population (beta-diversity). The second considers GMB composition and dysbiosis. Associations or correlations between dysbiosis and diseases are recalled and selected studies reviewed, mostly involving malnutrition, obesity, diabetes and inflammatory bowel disease (IBD). Examples of bacterial genomic markers of disease are given. References are provided to some recent, more specialized reviews in this vast area. The third section briefly reviews effects of gut bacteria on immune modulation, with a focus on innate immunity and inflammation. Finally, the last and main section covers gut bacteria metabolite chemistry and host gene regulation by bacterial metabolites.

## 2 Intestinal microbiome composition and homeostasis

The small intestine, in humans, has a length of 6–7 m and transit times of 3–5 h. With its enormous area of around 250 m^2^, it is the main site for nutrient absorption. The large intestine, or colon, downstream, has a length of around 1.5 m, an area of around 2 m^2^, and transit times of over 30 h. Physical and chemical differences between the two intestines (such as pH, oxygen levels, nutrient gradients, transit velocity and mucus thickness) lead to very different bacterial population densities. The vast majority of the microbiota live in the colon, at a population density of around 10^10^–10^12^ bacteria per milliliter, compared to just 10^4^–10^7^ per milliliter in the small intestine (23). There are also composition differences between the small and large intestinal microbiomes, indicated further on.

**Figure 1:**
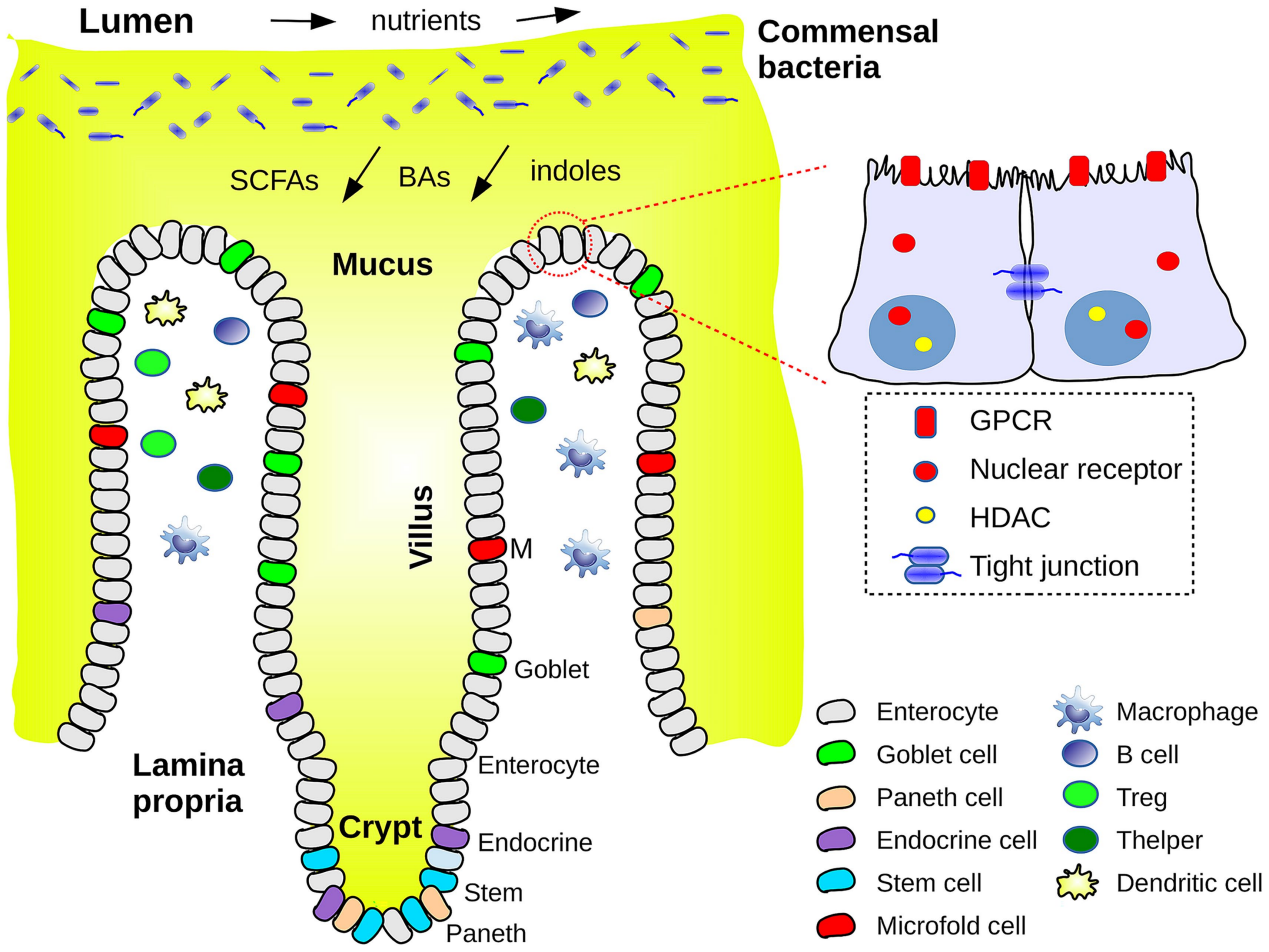
Schematic view of the interface between the intestinal cavity or lumen and the outer layers of the intestinal barrier or mucosa. Commensal bacteria reside in the outer layer of mucus. The flow of nutrients and of metabolites (SCFAs, bile acids, indole derivatives) is schematized. Epithelial and immune cell types are indicated. A closeup of two enterocytes shows a few chemosensory receptors, histone deacetylases (HDACs) and a tight junction.

The outer gut layer is the mucosa, which is exposed to the intestinal cavity or lumen. The mucosa and its commensal bacteria are schematized in Figure 1. As one moves from the intestinal cavity in toward the body interior, the first mucosa layer is a mucus layer, itself composed of two regions: an outer layer rich in bacteria and bacterial products (especially in the colon) and an inner layer with sparse bacteria and protective antibacterial peptides (e.g., defensins) (24, 25). Each mucus layer is a hydrated gel about 50–100 microns thick, composed mainly of the Mucin-2 protein, produced by goblet cells in the epithelium. Most of the long Mucin-2 polypeptide chain has a low sequence complexity, is heavily glycosylated, and is structurally disordered (both in humans and mice). Beneath the mucus is a coating of epithelial cells that is one cell thick. In a healthy host, the inner mucus layer creates a physical barrier avoiding direct contact of bacteria with the epithelium. Next come the lamina propria layer of connective tissue where immune cells reside, and a layer of smooth muscle, the Muscularis mucosae (furthest from the intestinal cavity). The mucosa surface is deeply corrugated, with millimeter clefts, filled with mucus and terminated by deep “crypts” (1). In the small intestine, the clefts alternate with millimeter extrusions, or “villi,” responsible for the small intestine’s enormous area. The interface between the gut and the lumen has similarities to some other biological interfaces, such as the coral-sea water interface and the root-soil interface of plants (26), where nutrients are absorbed and mucus secreted, along with defensive toxins.

Intestinal epithelial cells (IECs) are renewed every few days and contain different cell types, produced by stem cells at the base of the crypts (Figure 1). They include enterocytes to absorb nutrients and transport water and waste products, goblet cells responsible for mucus production, enteroendocrine cells for hormone secretion, Paneth cells for secretion of antimicrobial peptides and proteins, and microfold cells involved in antigen capture and presentation to immune cells (27). These cell types are present in both the small and large intestines, with somewhat different densities along the gut; for example, goblet cells and mucus production are more pronounced in the colon. IECs are linked to their neighbors by one of four types of junctions, including “tight junctions.” Junctions are formed by transmembrane proteins that bind, through their extracellular domains, to other junction proteins, within the same or a neighboring cell. Through their intracellular domains, they communicate with, and are often anchored to the actin cytoskeleton within. These junctions leave a gap or pore between neighboring cells, allowing penetration of water, electrolytes and small solutes (< 8 Å) (15).

The microbiome has been referred to as an “ecosystem on a leash” (26). Gut bacteria compete and cooperate with each other, while undergoing selective pressure from the host and providing and receiving benefits. Evolutionary theory provides some general information. For example, functional redundancy is expected to appear spontaneously; e.g., several microbes can produce similar molecules, and weak competitive interactions between microbes are expected to be most common (26). In this ecosystem, IECs communicate with the commensal bacteria to shape the composition and function of the microbial community. In return, commensals help strengthen the gut barrier and assist immune development (28). The full set of GMB functions can be thought of as a *functional microbiome*. They include energy harvesting and breaking down indigestible food. Since the gut is linked to the liver via the portal vein and the bile duct, resulting metabolites can join the general circulation and exert effects throughout the body.

Many gut bacteria cannot be cultivated in the lab, despite ongoing high-throughput efforts (29, 30). They mostly live in the oxygen-free colon, housed and fed by the outer mucus layer, in a mutualistic relation with their host and hundreds of other species, difficult to replicate in a Petri dish. However, metagenomics and high-throughput sequencing have transformed our ability to investigate the GMB, allowing not only a precise classification via 16S rRNA sequences, but the detection of spatial, temporal, and disease-related patterns (9). Thus, 3.3 million microbial genes in the human gut were cataloged in 2010 (2). Fetal microbiota were collected from 145 European women, and 453 gigabytes of sequence reads were processed (18.6 million genes) and compared to 2,382 reference bacterial genomes (31). Bacterial species whose relative abundances define a healthy GMB during the first 2 years of life were identified by applying a machine-learning approach to 16S rRNA data from monthly fecal samples in a cohort of healthy Bangladeshi children (32). The Human Microbiome Project Consortium (33) collected 4,788 specimens from 242 healthy adults, at 1 or 2 time points, from 18 or 15 body sites, including the gut. 16S rRNA sequences were determined, and marker sequences linked to particular bacterial species were identified. Based on all this data, the GMB is highly enriched in genes for energy production and metabolism.

Taxonomy classifies gut bacteria according to strain, species, genus, family, and phylum; hundreds of bacterial phyla exist. The typical adult GMB includes several hundred bacterial species (34) and thousands of strains. A minority are in the small intestine (23). The duodenum (in mice) contains mostly *Actinobacteria* and *Proteobacteria* (facultative anaerobes), the jejenum contains mostly *Bacillota*, and the ileum appears to mostly contain *Bacteroides, Clostridium, Enterobacteria, Enterococcus, Lactobacillus*, and *Veillonella*, which are facultative or obligate anaerobes. More details on regional GMB diversity are in a recent review (23) and a recent metagenomic study (35).

The vast majority of gut bacteria (and the most studied) are in the colon, which only tolerates obligate anaerobes. A few species of interest are in Table 1. A healthy human reference microbiome list and abundance profile (GutFeelingKB) were presented recently (36), listing 157 organisms (8 phyla, 38 families, 59 genera, and 109 species) that form the baseline adult GMB and can be used as controls for studies of dysbiosis. Most of the mammalian GMB is formed by the *Bacteroides* genus of Gram-negative, obligate anaerobes within the *Bacteroidota* phylum (formerly known as *Bacteroidetes*). The Gram-positive *Bacillota* phylum (formerly *Firmicutes*) is also abundant. In the gut of one human adult cohort, *Bacteroidota* and *Bacillota* represented 93% of 16S rRNA sequences (37). Three main variants or “enterotypes” were determined, based on the dominant genera (9). More recently, 8 bacterial phyla were found to be abundant in 1,000 healthy humans (38), with 126 abundant species: 31 *Bacteroidota*, 63 *Bacillota*, and 32 *Actinomycetota* (36). Each body habitat was characterized by a few signature taxa (33). Core, “housekeeping” gene functions were present in all habitats and enterotypes, but there was more variability for niche-specific pathways (33). For example, bacteria that produce SCFAs are mostly in the colon, whereas some lipid and indole transformations occur in the small intestine.

**Table 1:** Bacterial species and genera mentioned in main text.

Phylum	Species	Property of interest
*Bacillota* (or *Firmicutes; mostly Gram+*)	*Lactobacillus* spp.	Increased in obese subjects
*Bacteroidota* (or *Bacteroidetes; mostly Gram-*)	*Bacteroides fragilis*	Increased in obese subjects
*Bacteroidota*	*Bacteroides vulgatus*	Decreased in obese subjects
*Bacteroidota*	*Bifidobacterium* spp.	Decreased in obese subjects
*Bacillota*	*Clostridium* genus members	Reduced in T2D subjects
*Bacillota*	*Lactobacillus* spp.	Early colonization correlates with allergy decrease
*Bacillota*	*Clostridium*, *Eubacterium*, *Butyrivibro* genus members	Produce SCFAs at high levels in gut
*Bacillota*	*Blautia hydrogenotrophica*	Acetogen
*Bacillota*	All *Lachnospiraceae*	Remove 7-OH of deconjugated BAs to form DCA, LCA
*Bacillota*	All *Ruminococcaceae*	Remove 7-OH of deconjugated BAs to form DCA, LCA
*Bacillota*	*Clostridium bolteae*	Produce Bas conjugated to new AAs Phe, Leu, Tyr
*Bacteroidota*	*Bacteroides* genus members	Produce indole derivatives
*Bacteroidota*	*Bifidobacterium* genus members	Produce indole derivatives
*Bacillota*	*Lactobacillus* genus members	Produce indole derivatives
*Bacillota*	*Clostridium* genus members	Produce indole derivatives
*Bacillota*	*Lactobacillus reuteri*	Produce indole using tryptophanase
*Bacillota*	*Clostridium sporogenes*	Produce indole using tryptophanase
*Bacillota*	*Clostridium* genus members	Synthesize polyamines
*Bacillota*	*Lactobacillus* genus members	Reduced inflammasomes NLRP6,3 lead to reduction
*Bacteroidota*	*Prevotellaceae* family members	Reduced inflammasomes NLRP6,3 lead to increase

Maternal bacteria can shape a baby’s GMB through vaginal delivery and breastfeeding. Recently, mobile genetic elements from maternal bacteria were also found to contribute (39). The co-development of mother and infant GMBs was tracked from late pregnancy to 1 year of age using multi-omics data from 70 mother-infant pairs. Almost 1,000 genes were found to be transferred from bacterial species present in the mother to ones present in the infant; whether transfer occurred in the mother or the infant host could not be determined.

GMB changes over time were reviewed recently (4). Dominant GMB signatures and enterotypes are stable on the scale of months and years in healthy adults. Large changes can occur at specific development stages, such as puberty, pregnancy, old age, and the introduction of solid food in infancy. More generally, durable GMB changes can arise from changes in diet, disease, the use of antibiotics (9) or perturbations like stress or physical activity (4). For example, low intake of saturated fatty acids is a diet choice that is associated with increased microbial diversity (40).

## 3 Intestinal microbiome composition and dysbiosis

Commensal gut microbes participate in multiple functions and interact closely with our immune system. As a result, certain GMB disturbances are “dysbiotic,” and associated with pathologies (41). Considerable efforts have been made to identify patterns associated with specific diseases or lifestyles (36, 42–44) and develop therapeutic strategies. Experimental difficulties are numerous, including poor statistics (45) and lack of precision in host variables (27). Nevertheless, several strong associations have been demonstrated. An overview from 2012 listed 9 pathologies; an update listed 13 [Table 1 of (9, 43)], including allergies, gastric/colon cancer, obesity, inflammatory bowel disease (IBD), diabetes, anorexia and steatosis. Links have also been found to autism, anxiety and depression, reflecting the gut-brain axis (42). Transplanting specific GMB components from diseased mice into GF healthy mice showed that several of the diseases–obesity, colitis, metabolic adiposity–can be transmitted via the GMB (28). We review selected dysbiotic examples here.

Smith et al. considered links between the GMB and malnutrition–the leading cause of child mortality worldwide (46). A total of 317 Malawian twin pairs, either monozygotic or dizygotic, were followed for their first 3 years of life. Over time, half remained well-nourished while 43% became discordant for kwashiorkor, a form of severe acute malnutrition. Each discordant pair was treated with a ready-to-use therapeutic food. This produced a maturation of metabolic functions in kwashiorkor GMBs that regressed when therapeutic food was stopped. Fecal communities from several discordant pairs were transplanted into “gnotobiotic” mice (born in germ-free conditions). The combination of Malawian diet and a kwashiorkor microbiome in the donors produced weight loss in the mice, and perturbations in amino acid, carbohydrate, and intermediary metabolism, implicating the GMB as a causal factor in kwashiorkor. GMB alterations in malnourished Bangladeshi children were also observed (32). A machine-learning model was developed from healthy children, then applied to children with acute malnutrition. Acute malnutrition was found to be associated with significant GMB alterations, which were only partially countered by two widely-used nutritional interventions.

Two recent studies considered malnutrition in the context of cancer cachexia (or “wasting syndrome”). A cancer cachexia mouse model exhibited weight loss, muscle atrophy, and elevated inflammatory factors (47). Gut microbiota in the cachectic mice showed decreased diversity and imbalance relative to controls. Fourteen bacterial genera were identified as potential cachexia markers. Gene function prediction revealed changes in the functional GMB of the cachectic mice, especially in carbohydrate and lipid metabolism pathways. Gut alterations were also studied following broad-spectrum antibiotic treatment in the presence and absence of cancer cachexia, using a mouse model (48). Effects on the host were determined at the molecular level, by measuring the muscle proteome adaptations in response to bacterial depletion by antibiotics. Comparing healthy mice with and without antibiotic treatment, over 140 host proteins were differentially expressed. In cachectic mice, adaptations were blunted, with only 80 proteins differentially expressed (with and without antibiotics). The affected proteins were involved in processes such as glucose metabolism, oxidative stress response and muscle development, which all have known links to the microbiome.

There are also links between the GMB and weight loss or gain. Early studies investigated human fecal microbial composition in relation to obesity. Few consistent findings were observed and a meta-analysis showed that many of the studies were statistically underpowered (45). Nevertheless, some salient features emerge (9, 49). Turnbaugh et al. characterized fecal microbial communities of adult female monozygotic and dizygotic twin pairs (154 people in the United States) concordant for leanness or obesity, and their mothers, to determine how host genotype, adiposity, and environment influence the GMB and vice versa (50). Obesity was associated with phylum-level changes in the GMB, with an increased proportion of the *Bacillota* phylum relative to *Bacteriodota* and a decreased alpha-diversity. At the class level, obesity has been associated with increases in *Lactobacillus* spp. and *Bacteroides fragilis* and decreases in *Bacteroides vulgatus* and *Bifidobacterium* spp. (Table 1). Based on almost 2 million bacterial 16S rRNA sequences, the obese cohort had an altered representation of bacterial genes and metabolic pathways, with an increased capacity to harvest energy from food. Transplanting gut microbiota from normal to GF mice increased body fat (37). Two recent studies confirmed the phylum shift and reduced alpha-diversity associated with obesity (51, 52).

Associations with diabetes were revealed early (9). Cani et al. showed that exposure to high levels of lipopolysaccharide (LPS), a structural element of the membrane of Gram-negative bacteria, might lead to a higher occurrence of diabetes in mice (53). A metagenome-wide association study of gut microbiota in type 2 diabetes (T2D) was reported (54). Deep sequencing of the GMB DNA from 345 Chinese individuals (T2D or controls) was followed by taxonomic assignment and functional gene annotation. The proportion of the *Bacillota* phylum in the gut of T2D patients was significantly reduced (similar to obesity), as was the *Clostridia* class. T2D patients had a moderate GMB dysbiosis, a decrease of butyrate-producing bacteria, an increase in some opportunistic pathogens, and an enrichment of microbial functions for oxidative stress resistance. Around 60,000 bacterial genomic markers for T2D were identified. They differentiated between T2D cases and controls with a higher specificity than analyses based on human genome variation. A depletion of butyrate-producing bacteria in T2D patients was also seen more recently (45). These examples illustrate the potential of bacterial markers for host disease diagnosis or prediction.

The GMB was also characterized in European women evolving from normal glucose regulation to prediabetes and diabetes (31). Fecal microbiota were collected from 145 70 year-old European women with different glucose tolerance levels. 453 Gb of bacterial sequence reads were compared to 2,382 reference genomes. A “metagenomic cluster” was defined to be a group of bacterial genes consistently found together in the same individuals; 26 clusters differed between T2D and normal subjects. The association of clusters with host clinical markers was described, including body mass index and levels of cholesterol, triglycerides, lipoproteins, creatinine, tumor necrosis factor-alpha (TNFa), interleukin-2 and insulin. The clusters allowed effective T2D identification.

GMB associations with some other diseases were also detected (9). Thus, colonization with *Lactobacillus* spp. early in life is associated with decreased allergies (55), while the GMB has a higher diversity in Celiac’s disease patients than in healthy controls (56). Significant variations in GMB composition were seen in both the early and later stages of colorectal cancer (47), and models were developed that predict cancer progression based on the GMB signature (57, 58).

IBD groups several inflammatory conditions, the two main ones being Crohn’s disease and ulcerative colitis. On the host side, over 200 single nucleotide polymorphisms have been implicated in IBD, in host genes coding for NOD-like receptors, cytokines and antimicrobial proteins (15). One study in rodents used metagenomics, transcriptomics and metabolomics to show that in colitis, colon biological processes are primarily enriched for pathways linked to immunity (59). On the GMB side, links to IBD are related to the increased inflammation and to gut permeability. In a study with human cohorts, the metabolic profile in the stool was correlated with the level of calprotectin, a biomarker for inflammation severity; the higher the inflammation, the lower was the observed GMB alpha diversity (60). More generally, a long term overall dysbiosis is observed in IBD patients compared with controls, including lower microbial diversity (61–63). Over time the dysbiosis persists, with altered alpha and beta diversity, an increase in the *Bacteroidaceae* and a decrease in the *Prevotellaceae* families compared with healthy controls, who conserve a stable, healthy microbiota over 2 years (64). Another observation linking GMB and IBD is an imbalance in bacterial metabolites. Indeed, SCFA production was altered in a cohort of IBD patients (64), due to lower abundancies of specific bacteria such as *Faecalibacterium prauznitzii*, which produces butyrate and helps regulate the lymphocyte T cell balance (65). For BAs, 10 small to medium-size cross sectional studies (66) showed decreased primary BAs and increased secondary BAs.

Finally, in addition to dysbiosis and disease, changes of the GMB at certain ages and development stages may provide clues for early risk assessment of gut diseases. For example, old age has been linked with a reduced abundance of bacterial species that support SCFA production (67). The shift in gut bacteria at the sexual maturity stage and its relation with gut inflammation were explored in Sprague–Dawley rats (68); phylum shifts in the GMB were observed, with a *Bacillota*/*Bacteroidota* ratio that correlated with body mass.

## 4 Immune modulation and inflammation

Interactions with gut bacteria are essential for immune development and have been referred to as “conditioning” (69). Some background information is recalled here, whereas specific interactions involving bacterial metabolites are covered in the following section. The innate immune system contains Pattern-Recognition Receptors (PRRs) that detect microbial antigens by recognizing Pathogen-Associated Molecular Patterns, or PAMPs. PRRs include Toll-like membrane receptors (TLRs, some of which bind LPS), cytoplasmic NOD-like receptors (NLRs, some of which bind peptidoglycan), and lectins (which bind fungal PAMPs). NLRs include three subfamilies: NODs (nucleotide-binding oligomerization domain-containing proteins), NLRPs (Node-like receptor proteins), and the IPAF subfamily. When activated, TLRs and NLRs dimerize and transduce signals via the NF-κB and MAP kinase pathways. This promotes the production of innate immune cells like monocytes and macrophages, which secrete pro-inflammatory cytokines such as tumor necrosis factor-alpha, interferon-gamma, and various interleukins.

PRRs also participate in the formation of inflammasomes. These are large, cytoplasmic, multiprotein complexes that sense PAMPs. They cleave and activate caspase-1 in the canonical inflammatory pathway. Active caspase-1 can then cleave the pro-inflammatory cytokines interleukin-1 beta (IL-1β) and IL-18 into their active forms (70). Several types of inflammasomes, including the Node-like receptor protein family (e.g., NLRP1, NLRP3, NLRP6, NLRP12, NLRC4) interact with gut microbiota to maintain gut homeostasis, as illustrated in section 5 below. Inflammasome activation requires two signals: the first is an NF-κB-mediated signal for transcription of the NLRP3 and pro-IL-1β genes. The second can be viral, bacterial or mitochondrial DNA, a reactive oxygen species, a toxin, or excess cholesterol (71).

Interactions with commensal bacteria promote maturation of immune cells (9, 72). For example, bacterial metabolites stimulate B cell differentiation in order to produce immunoglobulin A and trigger formation of T-helper cells and regulatory T cells (Treg cells) (73); Treg cells play a key role in suppressing inflammation (12). Conversely, excessive hygiene is thought to contribute to immune pathologies such as allergies, with reduced levels of some anti-inflammatory cytokines (74). Another element of the gut immune system are glial cells, which colonize the intestinal mucosa after birth and mediate communication between the nervous and immune systems. Both the initial colonization and homeostasis of glial cells were shown to be regulated by the GMB (75).

Gut bacteria are a source of inflammatory molecules, such as flagellin, lipoproteins, LPS and peptidoglycan (PGN). Increased gut permeability and translocation of these molecules can then activate a chronic low-grade inflammation (24, 44, 76). Perturbation of the GMB can also create an inflammatory environment (15). Thus, GMB imbalances were shown to correlate with autoimmune diseases such as IBD (28); patients with symptomatic atherosclerosis had characteristic GMB changes (31), and the alteration of specific GMB strains progresses in parallel with liver steatosis (77, 78). In return, the inflammation machinery can act on the GMB. Thus, inflammasomes act as regulators of the GMB, since a deficiency in components of the inflammasomes NLRP6 and NLRP3 led to an altered, transmissible, colitogenic GMB (56). Examples where specific bacterial metabolites are known to influence or communicate with the immune system are considered in the next section.

## 5 Transformation of food products and metabolites by gut bacteria and impact on host gene regulation and physiology

Gut bacteria produce many essential metabolites. For example, they produce several amino acids (AAs) and consume others, and great differences were observed for the free AA content of the digestive tract between mice with and without a microbiome (79). Colonic bacteria also produce SCFAs, which can be uptaken into host cells by specialized membrane transporters. Gut bacteria synthesize vitamin B12, biotin (vitamin B7), folic acid (vitamin B9), and vitamin K2, although these are not necessarily all absorbed directly by human hosts, being mostly produced in the colon, downstream of the small intestine where most nutrient absorption takes place. Gut bacteria perform chemical transformation of bile acids that leads to secondary BAs for signaling and gene regulation. They perform indole chemistry that produces precursors to other important molecules, such as serotonin, melatonin, niacinamide, and vitamin B3. Bacteria also shed soluble PGN and LPS fragments; PGN is undetectable in the serum of GF mice, supporting the GMB as the origin of systemic PGN. The main GMB metabolites are considered below.

### 5.1 Generation of SCFAs and impact on host gene regulation

#### 5.1.1 Structure and origin of SCFAs

A fatty acid is a carboxylic acid with an aliphatic chain. SCFAs have just 2–4 carbons; the most common (and smallest) are acetate, propionate and butyrate. SCFAs have two sets of functions: as stand-alone chemicals and as signaling molecules. Thus, SCFAs are predominantly metabolized by enterocytes and hepatocytes, for which they act as combustibles (80). Specific anaerobic bacteria produce SCFAs at high levels in the colon by fermenting dietary fiber (67), in particular, members of the *Clostridium*, *Eubacterium*, and *Butyrivibrio* genera. The most abundant SCFA overall is acetate (50%), formed mainly by acetogens like *Blautia hydrogenotrophica* (12, 81) (Table 1), whereas butyrate is produced from acetate and lactate, and propionate is mainly produced from succinate (82) by *Bacteroidota*, *Bacillota*, and *Proteobacteria*. Details of the succinate-propionate, succinate-butyrate and succinate-acetate synthetic pathways were reviewed recently (82, 83). The short chain of SCFAs allows significant water solubility; the concentration of SCFAs is estimated to reach 50–100 mM in the colon. They are absorbed into colonic epithelial cells by two types of SCFA transporters: the monocarboxylate transporters MCT1 and MCT4 and the transporters SMCT1 and SMCT2 (81, 84), which are also widespread in other tissues.

Different SCFAs are distributed differently in the body. Butyrate mainly stays in the colon, serving as the main energy source for the IECs, or colonocytes (38, 67, 80, 85). Inside a colonocyte, butyrate can undergo one round of fatty acid beta-oxidation (86, 87), shedding two methylene groups and leading to one molecule of acetyl-coenzyme A, the entry point to the Krebs cycle. The concentrations of acetate and propionate in the colon are lower than butyrate, and propionate mainly functions in the liver (81). SCFA abundance also depends on dietary fiber intake and consumption of SCFA-rich foods (12). For example, a fiber-rich Mediterranean diet has been associated with increased intestinal SCFA levels (88). SCFA abundance was altered in several diseases (81). Thus, the concentration of butyrate in IBD is considerably lower than in healthy controls (27), while hypertension has been linked to a reduction in acetate- and butyrate-producing bacteria (45).

#### 5.1.2 Impact of SCFAs on host gene regulation

##### 5.1.2.1 Interactions mediated by G-protein coupled receptors, or GPCRs

**Figure 2:**
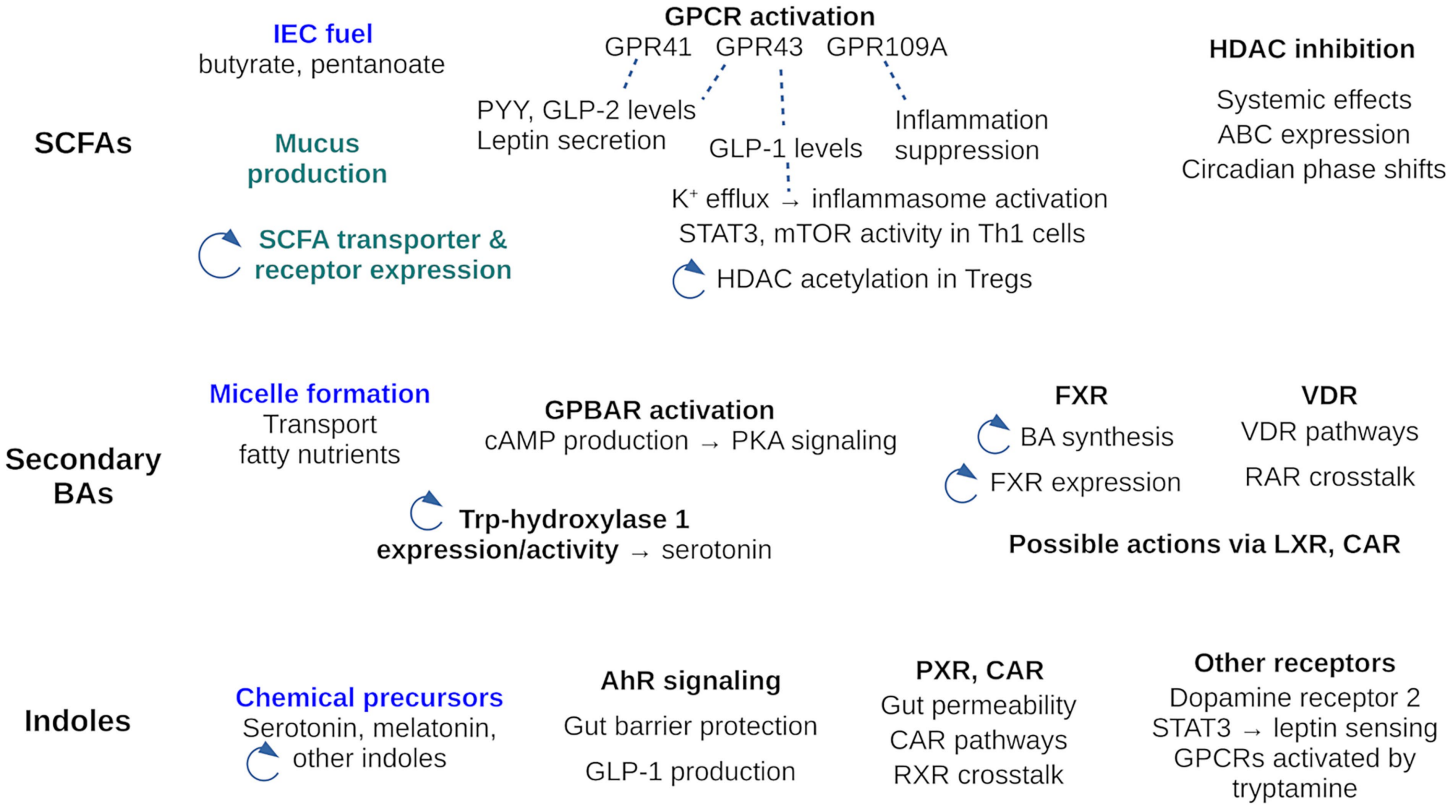
Activities of the three main GMB metabolite families: SCFAs, secondary BAs and indole. The activities cover those reviewed here, where the receptor mediating activity is known. Other activities, where the mechanism is unknown are not included in the Figure, with two exceptions (in green). Activities of the metabolites as stand-alone chemicals are in blue; all others are signaling activities. A circular arrow highlights activities that participate in a feedback loop. For example, SCFAs regulate expression of their own transporters; BAs influence the indole pool through Trp hydroxylase activity; SCFAs influence HDAC acetylation which modulates the level of HDAC inhibition by SCFAs.

In addition to being combustibles, SCFAs are important signaling molecules. Actions mediated by specific host receptors are reviewed below and summarized in Figure 2. SCFAs bind specifically to several host G-Protein Coupled Receptors (GPCRs) and act as agonists. GPCRs are membrane proteins present only in eukaryotes; over 750 have been identified or predicted in humans (89).^1^ They detect specific molecules outside the cell, whose binding induces a conformational change; this activates a target G-protein inside the cell and produces a response. Two GPCRs were shown to use SCFAs as agonists: GPR41 and GPR43. GPR43 (also known as Free Fatty Acid Receptor 2 or FFAR2) was shown to use formate, acetate, propionate, butyrate, isobutyrate, and pentanoate as agonists (90, 91), with a preference for propionate. GPR43 is expressed in leukocytes. GPR41 (or FFAR3) is homologous to GPR43, with 43% sequence identity in humans. It was shown to be activated by the same SCFA ligands (90, 91), with a preference for pentanoate. GPR41 is expressed in several tissues, including adipose tissue, the intestine, liver, pancreas, and skeletal muscle (45). 3-dimensional structures from cryo-electron microscopy exist for both receptors, including a GPR43 structure with bound acetate (PDB code 8J24) and a GPR41 structure with bound butyrate (PDB code 8J21). A third SCFA-activated GPCR is GPR109A (also known as Hydroxycarboxylic Acid Receptor2 or HCAR2; (92)). GPR109A is expressed by mature neutrophils in adipose tissue and spleen (93). It is activated by both butyrate and nicotinic acid (or niacin, a form of vitamin B3, another carboxylic acid). Niacin, like SCFAs, is produced by gut microbiota; it is known to suppress intestinal inflammation (92). There may well be other SCFA-activated GPCRs yet to be identified.

One effect of SCFA signaling is to promote epithelial barrier integrity (45). Thus, acetate binds to GPR43 and stimulates potassium efflux in epithelial cells, leading to activation of the NLRP3 inflammasome and production of interleukin-18 (IL-18), two pathways for promoting epithelial integrity (12). Butyrate was shown to directly induce Mucin-2 expression in human goblet cell lines (94). Butyrate also limits gut permeability by enhancing tight junction protein claudin-2 expression via an IL-10-receptor-dependent mechanism (27). Synaptopodin is another tight junction protein whose expression is induced by butyrate (12).

SCFAs were also found to affect appetite, through intestinal hormones, in a GPCR-dependent manner. They affected the glucagon-like peptides 1 and 2 (GLP-1, GLP-2) and the anorexic hormone peptide YY (PYY), which are all involved in appetite and the gut-brain axis (45). SCFAs increased GLP-1 plasma levels in rodents by a GPR43-dependent mechanism (95, 96), and increased PYY and GLP-2 levels in the circulation by activation of specific GPCRs, including GPR43 and GPR41 (97–99). In adipocytes, GPR41 activation by propionate stimulates secretion of leptin, the main hormone driving food intake (100), and promotes extracellular signal-regulated kinase 1 and 2 phosphorylation, leading to diverse responses (101).

Recent findings confirm that SCFAs are important immune modulators (102) and can regulate T cell differentiation and function (67). SCFA interaction with GPCRs leads to increased expression of IL-10 and Transforming Growth Factor-beta (TGF-β), decreased expression of macrophage and neutrophil pro-inflammatory cytokines, and blockade of T helper type 17 (Th17) cell differentiation (28, 103). SCFAs also interact with GPR43 to activate Signal Transducer and Activator of Transcription 3 (STAT3) and mammalian Target of Rapamycin (mTOR) pathways in T-helper 1 (Th1) cells; this upregulates the transcription factor B lymphocyte-induced maturation protein 1 (Blimp-1) (12). Finally, GPR109A, another SCFA target, inhibits NF-κB activation, modulating the production of pro-inflammatory cytokines. Thus, butyrate was shown to suppress LPS-induced NF-κB activation via GPR109A *in vitro*, whereas in monocytes butyrate induces expression of IL-10 (45).

##### 5.1.2.2 Interactions mediated by histone deacetylases, or HDACs

Another role of SCFAs is to inhibit histone deacetylases (HDACs), which help regulate host gene expression (Figure 2). HDACs deacetylate lysine side chains of histone proteins, previously acetylated by a histone acetyltransferase. Deacetylation restores the lysine positive charge and allows DNA to wrap more tightly around the histone. Conversely, HDAC downregulation favors histone acetylation, leading to decreased chromatin condensation and increased gene expression. HDAC inhibition is thus an important mechanism for host gene expression.

Several SCFAs, especially butyrate, are moderately potent HDAC inhibitors. Evidence for direct HDAC inhibition by an SCFA was given by Chang et al., who treated macrophages with n-butyrate and observed a down-regulation of proinflammatory mediators (104). Several lines of evidence suggested the observed effects were due to HDAC inhibition by butyrate. Waldecker et al. measured an inhibitory constant of 0.09 mM for butyrate, establishing a rather strong inhibition (105). Butyrate was also the most potent HDAC inhibitor in a whole-cell HeLa Mad 38-based reporter gene assay, while polyphenol metabolites and all other SCFAs tested were much less potent or completely inactive. Recently, HDAC inhibition by butyrate was shown to affect the expression of P-glycoprotein, an ATP-binding cassette (ABC) protein known as a multi-drug resistance transporter that protects cells via efflux of toxins and xenobiotics (106).

In addition to HDAC inhibition, butyrate and propionate can enhance histone acetylation through their metabolism to acetyl-CoA, an acetyl donor to histone acetyltransferases (107). Butyrate also regulates transcription via histone acylation, including propionylation and butyrylation (67, 108, 109).

In principle, SCFAs could have another effect on HDACs, activating GPCRs that influence HDAC expression or activity indirectly (81, 110). Possible evidence of an indirect, GPCR-mediated effect on HDAC activity came from the propionate treatment of colonic regulatory T cells (Tregs), which enhanced histone acetylation to an extent that depended on GPR43 expression (110). In addition, GPR41 activation was found to reduce the sustained elevation of histone acetylation induced by butyrate (111).

SCFAs were also shown to affect intestinal epithelial circadian rhythms by an HDAC-dependent mechanism (112). Sterile filtrates from four bacterial species within a defined murine GMB induced profound, concentration-dependent circadian phase shifts in cultured enteroids. The shifts could be traced to SCFAs through a machine learning approach. Pharmacologic HDAC inhibitors yielded similar effects.

Many other systemic effects have been associated with SCFAs, without a precise determination of the signaling pathways involved. In one study, SCFAs were found to participate in a feedback loop, by influencing the expression of colonic SCFA transporters and receptors, through an indirect effect associated with the GMB (113). A second study examined skeletal muscle atrophy in mice with and without a GMB (114). The germ-free mice had a reduced expression of genes associated with skeletal muscle growth and mitochondrial function, and an altered expression of myogenin, a gene downstream of the HDAC pathway.

### 5.2 Effect of gut bacteria on bile acids and impact on host gene regulation

#### 5.2.1 Structure and origin of BAs

**Figure 3:**
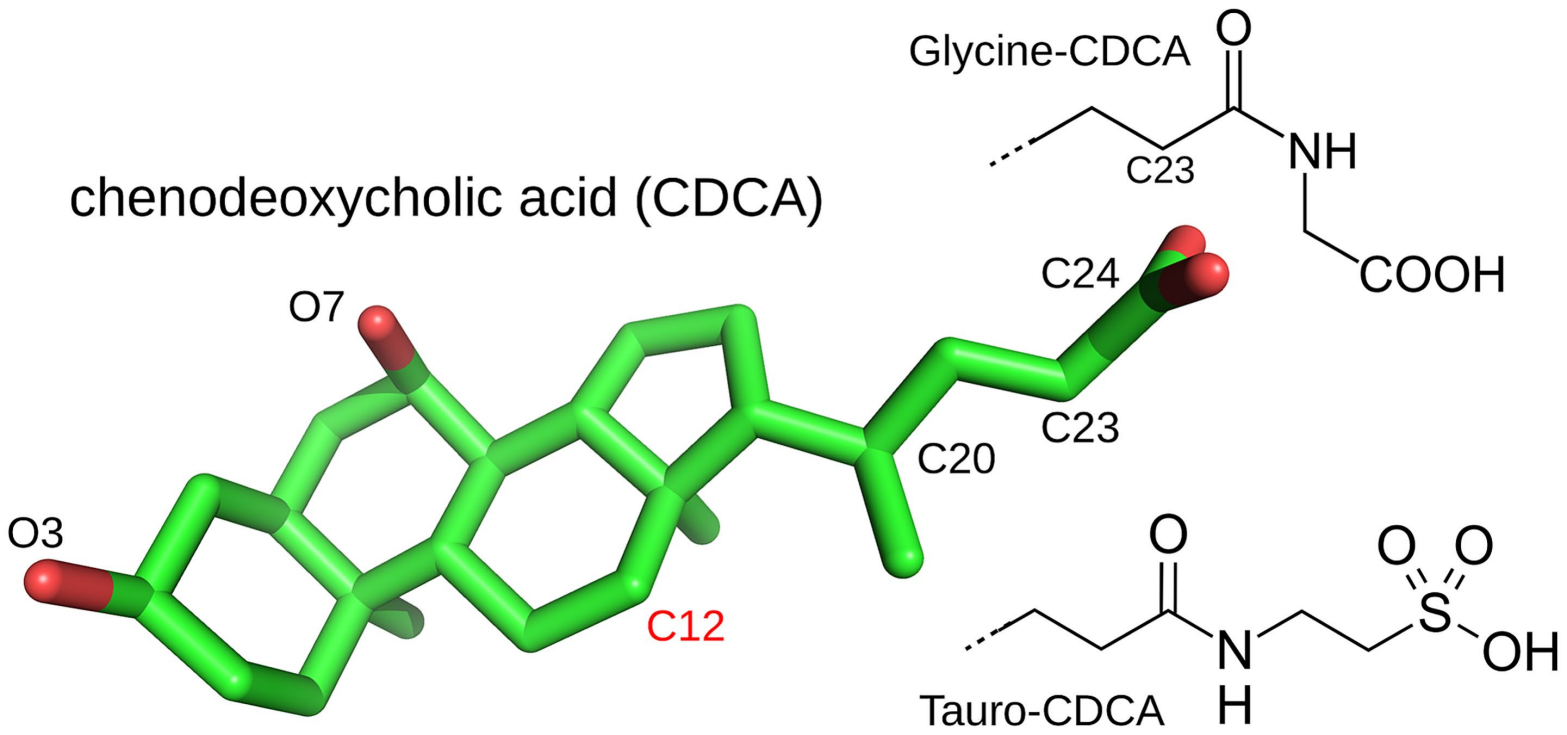
The bile acid chenodeoxycholic acid (CDCA) and its glycine and tauro conjugates. Cholic acid (CA) differs by an additional hydroxyl on C12. CDCA is from the PDB structure 6HL1 and rendered with Pymol (Schrodinger, LLC, The PyMOL Molecular Graphics System).

BAs are steroid alcohols, whose chemical structure is shown in Figure 3. They are end products of cholesterol metabolism, named for their carboxylic acid substituent at position 23. “Primary” BAs, such as cholic acid (CA), are synthesized in the liver through cholesterol oxidation, then stored in the gall bladder and secreted into the small intestine after meals through bile flow (115, 116). Their carboxylic group has a rather high pKa of around 5 and enhances their solubility only moderately in the acidic duodenum (pH of 3–5). Thus, most BAs are *conjugated* in the liver to either glycine (predominant in humans) or taurine (predominant in mice) via bile acid-CoA synthetase (BACS) and bile acid-CoA amino acid N-acyltransferase (BAAT). This downshifts their pKa and increases their ionic fraction and “salty” character. They can then form micelles and transport weakly-soluble fatty nutrients.

In the ileum (at the end of the small intestine), most BAs are reabsorbed and transported back to the liver. Unabsorbed BAs (5–10%) are further modified in the colon by gut bacteria to form “secondary” BAs. Thus, hydrolysis of the amide bond (at position 24) by the bacterial BA hydrolase *deconjugates* the BA and enables further microbial transformation, including dihydroxylation, oxidation, epimerization of hydroxyl groups, and reconjugation of new AAs to introduce structural diversity (12). All members of the *Lachnospiraceae* and *Ruminococcaceae* families strip away the 7-hydroxyl of deconjugated BAs to generate deoxycholic acid (DCA) and lithocholic acid (LCA), the most common secondary BAs and fat emulsifiers. Gut bacteria also use hydroxysteroid dehydrogenases to oxidize the hydroxyl groups at ring positions 3, 7 or 12 and produce oxo or keto BAs (115). Recently, new secondary BAs were discovered, conjugated to the AAs phenylalanine, leucine and tyrosine by *Clostridium bolteae* (117). These AAs are larger and more hydrophobic than glycine and taurine, altering BA binding and emulsifying properties. The role of BAs as signaling molecules is considered next.

#### 5.2.2 Impact of bacterially-modified BAs on host gene regulation

##### 5.2.2.1 Interactions mediated by nuclear receptors

Both primary and secondary BAs have emerged as signaling molecules that activate multiple receptors and influence multiple aspects of host physiology (14, 84, 118) including lipid, glucose, steroid, and energy metabolism. Signaling interactions where the specific receptor protein has been identified are summarized in Figure 2. The G protein-coupled receptor GPBAR1, also known as Takeda G protein receptor 5 is targeted by several BAs (119, 120), including secondary BAs. In response to BA activation, it induces cAMP production and activates protein kinase A signaling pathways. BAs have also been shown to inhibit the NLPR3 inflammasome (12). Secondary BAs may help regulate gut-derived serotonin production by increasing the expression and activity of tryptophan hydroxylase 1 (TPH1), the rate-limiting enzyme for mucosal serotonin synthesis (45). Another physiological effect was the increased level of unconjugated BAs seen in IBD patients (115, 121), although the precise IBD/BA mode of association has not been elucidated.

However, the main receptors targeted by BAs are nuclear receptors. The first is the farnesoid X receptor (FXR), also known as the BA Receptor (BAR) (122). The main ligand for FXR is chenodeoxycholic acid (CDCA), followed by CA, DCA, and lithocholic acid (LCA) (84). A crystal structure of the FXR: CDCA complex was determined recently (123), revealing a functional interaction. Resulting physiological effects include antimicrobial effects via FXR-induced antimicrobial peptide production (115). Another effect is the negative feedback control of BA synthesis (116). Indeed, FXR regulates expression of two key enzymes for BA synthesis, and tauro-conjugated beta- and alpha-muricholic acids were identified as FXR antagonists (116). Another study using FXR-deficient mice showed that the GMB regulates expression in the liver of several key proteins for BA synthesis, by FXR-dependent mechanisms (116).

Effects on muscle physiology were also identified. Metabolomics in the liver of GF mice revealed a higher taurine and tauro-conjugated BA level than in control mice (114). Treatment of adult mice with a cocktail of five antibiotics designed to deplete the full spectrum of gut bacteria changed the digestive BA profile, with more primary than secondary BAs in the ileum of the treated mice and a decrease in *fxr* gene expression in the ileum (124).

The secondary bile acid LCA is also a weak physiological ligand of another nuclear receptor, the vitamin D receptor (VDR) (125). BAs are chemically very different from vitamin D, and they bind 5–6 log units less strongly to VDR. Nevertheless, *in vivo* experiments showed that LCA derivatives effectively induce VDR activation (125). VDR heterodimerizes with the retinoic acid receptor (RAR), suggesting cross-talk with other signaling pathways.

Effects of the GMB on the circadian rhythm of the liver were linked to several nuclear receptors (126), some activated by cholesterol analogs and possibly BAs. Depletion by antibiotic treatment led to changes in circadian gene expression and corticosterone production in the gut, with a hypercorticosterolism that caused hyperglycemia and insulin resistance. The activity of several nuclear receptors was affected, including PXR, CAR (detoxification), LXRα and PPARα (lipid metabolism); the expression of their target genes was strongly influenced. PXR and PPAR are activated by the bacterial metabolites indole and vitamin B3 (nicotinic acid), while LXR and CAR are activated by cholesterol and analogs.

### 5.3 Effect of gut bacteria on indole derivatives and impact on host gene regulation

#### 5.3.1 Structure and origin of indole derivatives

Human enzymes do not synthesize tryptophan (Trp), making it one of the “essential” AAs obtained from dietary proteins [reviewed by (127, 128)]. Indole and its derivatives are then derived from the catabolism of Trp in the small intestine by gut bacteria having the tryptophanase enzyme, such as *Lactobacillus reuteri* and *Clostridium sporogenes* (27). The absence of indole derivatives in GF mice indicates the involvement of microbiota in their production (12). Trp-consuming bacteria that produce indole derivatives include anaerobes, *Bacteroides*, *Clostridium*, *Bifidobacterium*, and *Lactobacillus*. Resulting derivatives include indole, indole-acetic acid and indole-propionic acid. By way of these derivatives, Trp is a precursor in the synthesis of serotonin, melatonin, niacinamide, and vitamin B3. For example, bacteria in the gut microbiota were shown to mediate the production of serotonin by promoting Trp digestion in host colonic enterochromaffin cells (12, 100).

#### 5.3.2 Impact of indole derivatives on host gene regulation

Trp and indole derivatives also have signaling activities, some discovered recently, summarized in Figure 2. They are well-known agonists of the aryl hydrocarbon receptor (AhR) (12, 129), which belongs to the family of basic helix–loop–helix transcription factors. AhR is widely expressed in host immune cells and in the liver, and is involved in various innate and adaptive immune responses when activated by endogenous ligands and xenobiotics. Indole and its derivatives help support intestinal homeostasis by activating AhR to protect the intestinal tight junctional barrier (27). Supplementation of *Lactobacillus reuteri*, a high producer of indole derivatives, to mice fed on a high fat diet restored AhR-dependent metabolic homeostasis, including intestinal barrier function and GLP-1 production (12). In IBD, AhR expression is downregulated, together with Trp metabolites (73). Trp metabolites derived from the microbiome were also found to modulate white adipose tissue inflammation in obese patients, via the miR-181 family of microRNAs (28).

Tryptamine is known to activate several GPCRs targeted by serotonin (130), participating in the gut-brain axis. Recently, Trp metabolites were found to activate the neurotransmitter Dopamine Receptor 2, expressed in the intestinal epithelium (131). Indole 3-propionic acid was found to bind the STAT3 transcription factor, promote its phosphorylation and nuclear translocation (132), and act via STAT3 as a leptin sensitizer.

Indole 3-propionic acid is also a ligand for the epithelial pregnane X receptor (PXR), also known as the steroid and xenobiotic sensing receptor. PXR is a nuclear receptor known to be associated with gut permeability (27). It regulates transcription of the cytochrome P450 gene CYP3A4, by binding to the CYP3A4 promoter as a heterodimer with the 9-cis retinoic acid receptor RXR. Very recently, several diindole compounds were found to bind to another nuclear receptor, the constitutive androstane receptor (CAR), as agonists with nanomolar affinities (133). They were shown to activate CAR and upregulate target genes in human hepatocytes.

### 5.4 Other metabolites and their impact

Trimethylamine N-oxide (TMAO or (CH_3_)_3_NO) is metabolized from nutrients in food (such as phosphatidylcholine and choline) by intestinal bacterial enzymes (such as the choline trimethylamine-lyase CutC and the carnitine oxygenase CntA), then further metabolized by the host enzyme flavin-containing monooxygenase 3 in the liver (84). In humans, TMAO activates the NLRP3 inflammasome, as well as NF-κB and MAPK signaling in vascular smooth muscle cells and endothelial cells, triggering pro-inflammatory gene expression and endothelial adhesions (12); this promotes atherosclerosis and thrombosis (134, 135). Increased TMAO levels are also associated with a lowering of BAs, since TMAO inhibits CYP27A1 and CYP7A1, two key enzymes that drive BA metabolism (136).

Vitamin B12, the most complex of vitamins, is only synthesized by bacteria and archaea. It is a required cofactor for many enzymes, and uses a co-bound cobalt ion to function (137). In humans, it is produced in the colon, but excreted before it can be absorbed. Other animals do absorb bacterial vitamin B12, such as ruminants, where bacterial fermentation happens in the “foregut,” and animals like rabbits that reingest their own excrement. The bacterial vitamin can then reach human hosts that consume these animals.

Polyamines such as spermidine and spermine are synthesized by gut bacteria such as *Clostridia* (100). They are organic cations needed for synthesis of DNA, RNA and proteins. Polyamines also act as reactive oxygen species scavengers, acid tolerance factors, chemical chaperones, and regulators of the stress response (100).

PGNs secreted by *Lactobacillus* and *Bifidobacterium* enhanced the expression of host tight junction proteins, including claudins, occludin and ZO-1, and improved the integrity of the gut barrier via TLR2 signaling (15, 27). GMB-derived PGNs promoted regeneration of Lgr5 + stem cells in the mouse intestinal crypt via the NOD2-dependent pathway.

Much of the metabolic content of gut bacteria is formed of lipids, which represent for example 10% of the dry weight of an *E. coli* cell. Gut bacteria play an important role in digesting and transforming the dietary lipids of their hosts; associated microbiome enzymes were reviewed recently (138). In addition to transforming existing lipids, gut bacteria can also synthesize a wide variety of lipids (139). For example, potential sulfonolipid biosynthetic genes were found recently in several bacteria (140); these lipids are found in the outer membrane of some Gram-negative bacteria in the *Bacteroidota* phylum (141). Many lipids, like prostaglandin, act as signaling molecules that can be sensed by host Pattern Recognition Receptors, such as TLRs and NLRs and by G-protein-coupled receptors (142, 143).

## 6 Conclusion

Links between the GMB and host pathophysiology continue to emerge at a rapid pace. Many studies focus on correlations between the GMB and disease, and explore possible therapeutic strategies. Major efforts are underway to identify microbiome DNA sequence patterns associated with specific diseases. Machine learning models can already predict colorectal cancer progression based on the host microbiome signature (57, 58), and GMB metagenomic clusters allow effective T2D identification (31). Going beyond diagnosis, GMB transplants and probiotics have therapeutic potential. Fecal microbiota transplantation is already approved by the Food and Drug Administration for *Clostridium Difficile* infection, and many clinical trials are interested in GMB transplantation against ulcerative colitis and Crohn’s disease (28). However, recent meta-analysis considering 550 patients across 12 studies shows a need for precaution due to biosecurity (144).

Here, we mainly focused on basic mechanisms of bacteria-host interactions and their actors, illustrated by some classic studies of the last decade or so, along with more recent studies. Many previous review articles were referenced, which should allow readers to go deeper into selected areas of interest. The complexity of signaling pathways and systemic effects often obscures the precise mechanisms and causal relations. Many experimental difficulties and questions remain. Bacterial populations vary between human subjects, depending on ethnic group, gender, age, nutrition, genetics, and other factors, and relevant experimental cohorts are limited and costly. Thus, some association studies were statistically underpowered (45), while probiotic clinical trials can suffer from lack of precision in host variables. Protocols for stool sample collection and preservation remain diverse (145). In addition, the dosage and timing of bacterial metabolites varies between spatial niches in the gut of a single host, in ways that have only begun to be characterized (69). While several diseases are accompanied by GMB dysbiosis, changes to the *functional microbiome* are harder to characterize, and the role of dysbiosis as cause or effect is often uncertain. Can one act on the microbiome, through depletion and transplantation, or by administering or stimulating production of antibodies against specific bacterial antigens? Can one attenuate or prevent obesity, say, by acting on the microbiome? When the GMB is reconstituted after depletion by antibiotics, what role does the prior conditioning of the immune system play? Progress and mechanistic understanding will depend on the accumulation of far more data on animal and human cohorts.

Molecular actors also need to be better characterized. Thus, while three GPCRs are known to be activated by bacterial metabolites (SCFAs), biochemical and structural data such as agonist binding constants and crystal structures remain sparse. Other GPCRs activated by SCFAs and other metabolites probably exist and remain to be identified. Similarly, despite recent efforts to screen nuclear receptors against large libraries of small molecules, responses to many bacterial metabolites are still uncharacterized. PXR and PPAR are activated by the bacterial metabolites indole and vitamin B3, and LXR and CAR are activated by cholesterol and analogs. However, related metabolites may also activate these and other nuclear receptors, and cross-talk due to hetero-dimerization of receptors is not fully characterized. Analysis of 3D structures of nuclear receptors in complex with putative agonists could allow hypothesis testing. In addition to the host proteins involved, metabolites are not fully characterized; for example, most lipids in the stool are still unannotated and have unknown functions (139). Even more information is lacking on the interactions between metabolites; for example, interactions between TMAO and BAs were detected only recently (136).

Fortunately, experimental methods for metagenomics and high-throughput sequencing continue to advance rapidly, and structural biology has undergone major breakthroughs. High-resolution cryo-electron microscopy can now reveal structures of GPCRs with bound ligands as small as acetate and butyrate, providing evidence for functional interactions. Homology-based structure prediction with new deep learning tools such as Alphafold2 has allowed 3D models to be produced for millions of proteins, including all known nuclear receptors, GPCRs, and HDACs, and all known bacterial proteins. This opens the way to predict interactions between antibodies and putative bacterial antigens, for example. Animal models are also progressing; for example, animals with a more humanized metabolite pool have begun to appear (45). Synthetic biology continues to advance rapidly, and has begun to allow controlled studies, through the construction of bacteria that can titrate specific gut metabolites, such as hydrogen sulfide [a *gaseous* metabolite whose role in gut diseases is debated; (146)] and others (147, 148). Overall, it is an exciting time for understanding the GMB and advancing its therapeutic potential.

## Footnotes

^1^ https://www.uniprot.org

## Glossary

AA: amino acid
AhR: aryl hydrocarbon receptor
BA: bile acid
CA: cholic acid
CDCA: chenodeoxycholic acid
DCA: deoxycholic acid
FXR: farnesoid X receptor
GF: germ free
GLP: glucagon-like peptide
GMB: gut microbiome
GPCR: G-protein-coupled receptor
HDAC: histone deacetylase
IBD: inflammatory bowel disease
IEC: intestinal epithelial cell
IL: interleukin
LCA: lithocholic acid
LPS: lipopolysaccharide
NLR: NOD-like receptor
NLRP: Node-like receptor protein
NOD: nucleotide-binding oligomerization domain-containing protein
PAMP: pathogen-associated molecular pattern
PGN: peptidoglycan
PRR: pattern recognition receptor
SCFA: short chain fatty acid
T2D: type 2 diabetes
TLR: Toll-like receptor
TMAO: trimethyl N-oxide
Treg: regulatory T cells
Trp: tryptophan
VDR: vitamin D receptor
